# A Text-Book of the Diseases of Women

**Published:** 1895-01

**Authors:** 


A TEXT-BOOK OF THE DISEASES OF WOMEN. By Henry J. Garrigues, A. M., M. D., Professor of Obstetrics in the New York Post-Graduate Medical School and Hospital; Gynæcologist to St. Mark's Hospital, New York; Gynæcologist to the German Dispensary, New York; Consulting Obstetrician to New York Infant Asylum; Obstetrical Surgeon to the New York Maternity Hospital; Fellow of the American Gynæcological Society; Fellow of the New York Academy of Medicine, etc., etc. Illustrated with Three Hundred and Ten Engravings and Colored Plates. Price, Cloth, $4.00; Sheep, $5.00. Philadelphia: W. B. Saunders, 925 Walnut Street. 1894.

Professor Garrigues' "Text-Book of the Diseases of Women" is one of the best books on this subject which has appeared. It is distinctively American in its teachings, the author giving preference always to the methods employed by our American gynæcologists, rendering the book of far more value to students and practitioners than those written by foreign authors. The book is conveniently and well arranged. It is divided into a general and a special part. Special attention is paid to the anatomy of the female organs of generation, and much valuable information and many practical suggestions are here found, for which you may look elsewhere in vain. Theories and obsolete methods have little mention here—everything being practical and up-to-date. In the diagnosing and treatment of gynæcological cases, there is much of detail which will prove invaluable to those who have not the opportunities of hospital or post-graduate training. The book is written by one who, as teacher in college and hospital, realizes the importance of attention to detail and a thorough understanding of minor, but often essential points.

Prof. Garrigues is a man of distinguished ability. He is acknowledged not only as one of the leading specialists of America, but as second to none as teacher and author.

